# Novel phenotypes of immune-mediated necrotizing myopathy identified independent of myositis-specific antibody specificity that improve prognostic stratification

**DOI:** 10.3389/fimmu.2026.1821033

**Published:** 2026-05-14

**Authors:** Xiaojing Wei, Hui Sun, Zhidan Pang, Na Bai, Tiantian Guo, Changpu Nie, Xuan Yang, Zhen Lu, Liye Bao, Jing Miao, Xuefan Yu

**Affiliations:** 1 Department of Neurology and Neuroscience Center, The First Affiliated Hospital of Jilin University, Changchun, Jilin, China; 2 Center for Rare Diseases, The First Hospital of Jilin University, Jilin University, Changchun, China; 3 Department of Electrophysiology, The First Affiliated Hospital of University of Science and Technology of China, Hefei, Anhui, China; 4 Department of Emergency, Changchun Hospital of Traditional Chinese Medicine, Changchun, China

**Keywords:** clustering analysis, distinct phenotype, immune-mediated necrotizing myopathy, prognosis, proteomics

## Abstract

**Aim:**

To systematically investigate the clinical characteristics and prognosis of patients with immune-mediated necrotizing myopathy (IMNM), and to explore the proteomic landscape associated with different clinical phenotypes.

**Methods:**

A total of 133 IMNM patients were enrolled in this retrospective study. The clinical features and outcomes were compared across three subgroups: anti-HMGCR-positive, anti-SRP-positive, and seronegative patients. Unsupervised machine learning algorithms were applied to cluster patients independently of antibody status. Label-free data-independent acquisition quantitative proteomics was performed on samples from clustered IMNM patients and noninflammatory control subjects.

**Results:**

No significant differences were observed in the overall prognosis among the three antibody-based subtypes. Unsupervised machine learning, independent of myositis-specific antibodies (MSA) status, identified three distinct phenotypes with unique clinical presentations and prognoses. Phenotype 1 (56.4%) corresponded to patients with muscle weakness and a more favorable prognosis. Phenotype 2 (10.5%) was characterized by the highest creatine kinase and lactate dehydrogenase levels, with an intermediate prognosis. Phenotype 3 (33.1%) was marked by a high incidence of ILD and a high mortality rate. Proteomic profiling revealed that these phenotypes exhibited distinct protein expression patterns and associated biological processes.

**Conclusions:**

MSA are not effective for stratifying IMNM patients into subgroups that are more homogeneous with respect to ILD incidence and prognosis. Novel IMNM phenotypes identified independent of MSA status differ significantly in clinical outcomes and proteomic signatures, providing fresh insights into the pathogenic mechanisms underlying IMNM.

## 1 Introduction

Immune-mediated necrotizing myopathy (IMNM) represents a distinct clinical subset of idiopathic inflammatory myopathies. It is characterized by severe proximal muscle weakness, markedly elevated serum creatine kinase (CK) levels, and predominant muscle fiber necrosis accompanied by sparse or mild lymphocytic infiltrates (1, 2). The disease entity was first proposed by the European Neuromuscular Centre in 2003 and further subclassified in 2016 to include anti-3-hydroxy-3-methylglutaryl coenzyme A reductase (HMGCR) antibody-positive IMNM, anti-signal recognition particle (SRP) antibody-positive IMNM and seronegative IMNM.

Although anti-HMGCR, anti-SRP, and seronegative IMNM share common clinical presentations, emerging evidence highlights distinct features across these subtypes: severe limb/neck weakness, dysphagia, muscle atrophy, and cardiorespiratory involvement (more prevalent in anti-SRP IMNM) (3–10); associated connective tissue disorders (predominant in seronegative IMNM) (11); subtype-specific immunogenetic risk factors (e.g., HLA-DRB1*11:01 exclusive to anti-HMGCR IMNM); and differential outcomes (e.g., poorer prognosis in anti-SRP IMNM compared with anti-HMGCR IMNM) (5, 12, 13). These findings collectively underscore the inherent heterogeneity of IMNM.

Previous studies study, including our earlier work, have evaluated IMNM outcomes based on serological or pathological stratification; however, no statistically significant differences in prognosis were identified among different myositis-specific autoantibody (MSA) subgroups (3, 5, 11, 14–16). In line with these reports, we also failed to detect prognostic variations across the three serological groups in our study. Thus, it is essential to identify IMNM subgroups with shared clinical and biological characteristics from multiple perspectives.

Given that the relationship between antibody subtypes and prognosis remains incompletely understood. Therefore, we conducted this follow-up study with two primary objectives: first, to clarify the clinical and prognostic disparities among IMNM subgroups stratified by antibody specificity; second, to explore a more effective classification approach that can categorize IMNM patients into subtypes with greater homogeneity in clinical manifestations, long-term prognosis and underlying pathophysiology.

## 2 Materials and methods

### 2.1 Study participants and ethics review

A total of 143 patients diagnosed with IMNM at the First Affiliated Hospital of Jilin University between January 2015 and December 2024 were enrolled retrospectively. The diagnosis of IMNM was based on the 224th ENMC workshop criteria and further verified retrospectively by two expert neurologists (17). Ten participants with missing critical laboratory results were excluded, resulting in a final study cohort of 133 patients. Given the retrospective nature of the study and the anonymous use of patient data extracted from medical records, written informed consent was waived, and verbal consent was obtained from all patients through telephone follow-up before accessing their medical records. In addition, written informed consent was obtained from all patients who underwent proteomic testing, in accordance with the relevant requirement. All data including demographic information, clinical manifestations, and laboratory tests results were collected retrospectively from medical records of the initial hospital admission. Survival status and radiographic features were confirmed through a combination of medical record reviews and telephone interviews. All follow-up assessments were completed by November 2025, with each participant’s status documented at the final follow-up visit or at the time of death.

### 2.2 Definition of the variables

Blood and muscle samples for laboratory testing were collected from participants during their initial hospital admission. All clinical manifestations, laboratory results, and complications documented in this retrospective study were corresponded to the time of blood collection. Interstitial lung disease (ILD) was confirmed through a comprehensive evaluation, incorporating respiratory symptoms, high-resolution computed tomography (HRCT) findings, and pulmonary function test (PFT) results as a reference (18). The radiological findings were grouped into four main HRCT patterns by two senior radiologists: non-specific interstitial pneumonia pattern (NSIP), organizing pneumonia pattern (OP), NSIP/OP overlap pattern, and usual interstitial pneumonia-like pattern (UIP). Rapidly progressive ILD (RPILD) was defined as the presence of rapidly progressive dyspnea and hypoxemia in conjunction with deteriorating interstitial lung abnormalities on imaging, occurring within 3 months after the onset of respiratory manifestations (19). Muscle weakness was defined as muscle strength grade < 5 according to the British Medical Research Council Muscle Strength Scale (20). Respiratory failure was diagnosed based on prominent respiratory symptoms, mainly cough and dyspnea, with arterial blood gas analysis confirming either PaO_2_ ≤60 mm Hg without oxygen supplementation or a PaO_2_/fraction of inspired oxygen ≤300 mm Hg with supplemental oxygen (21). Pulmonary infections were identified by a comprehensive analysis of the clinical presentations, imaging results, and positive microbial tests.

### 2.3 Detection of MSA and myositis-associated antibodies

Anti-SRP, anti-Ku, anti-PM-Scl 100, anti-PM-Scl75 and anti-Ro-52 were tested using line immunoassay (EUROLINE, Luebeck, Germany). The result was considered weak positive if the band showed an intensity of 25–75, and an intensity greater than 75 was considered strong positive. Patients with weakly positive antibodies were confirmed by repeated testing. Anti-HMGCR antibodies were detected via ELISA according to the manufacturer’s instructions (Inova Diagnostics, San Diego, CA, USA).

### 2.4 Protein extraction and mass spectrometry data analysis

A total of 9 IMNM patients with active disease at initial admission and 3 noninflammatory controls (CTRLs) were recruited for protein extraction and MS analysis. Protocols for muscle tissue protein extraction and subsequent bioinformatics analyses, including differential protein expression profiling, weighted gene co-expression network analysis (WGCNA) (22), Gene Ontology (GO) enrichment analysis, and core protein screening, are described in detail in the **Supplementary Materials**.

### 2.5 Statistical analysis

Data analysis and visualization were conducted using IBM SPSS v27.0 and R software (v4.5.2). Quantitative data were presented as means (standard deviation, SD) or medians (interquartile range, IQR), depending on the data distribution pattern. Qualitative variables were summarized as counts (percentages). For comparisons of continuous variables across multiple groups, analysis of variance (ANOVA) or the Kruskal-Wallis test was applied, with the choice determined by data distribution characteristics. Dunnett’s or Bonferroni *post hoc* tests were performed for pairwise comparisons when appropriate. The chi-square (χ²) test and Fisher’s exact test were used to compare categorical variable frequencies among multiple groups. Survival outcomes were analyzed using Kaplan-Meier curves, with group comparisons performed via the log-rank test. In the survival analysis, patients who were lost to follow−up were treated as censored observations. Specifically, they were neither classified as deceased nor excluded; rather, their censoring time was defined as the date of the last available follow−up, at which they were considered alive and event−free. This approach is consistent with standard right−censoring methodology. Unsupervised machine learning algorithms were employed to classify IMNM patients. Least absolute shrinkage and selection operator (LASSO) regression was conducted to identify the most relevant features for each Cluster. Additionally, a predictive model was developed using the classification and regression tree (CART) algorithm. Detailed protocols for the unsupervised statistical workflow, LASSO regression, and CART algorithm are provided in the online **Supplementary Methods**. All statistical tests were two-tailed, and a *P* < 0.05 was considered statistically significant.

## 3 Results

### 3.1 Clinical characteristics of IMNM patients stratified by MSA specificity

In this cohort of 133 IMNM patients, 60 patients (45.1%) were anti-SRP-positive IMNM, 38 patients (28.6%) were anti-HMGCR-positive IMNM, and 35 patients (26.3%) were seronegative. The general clinical characteristics and intergroup comparisons are summarized in **Table 1**. Among all patients, 92 patients (69.2%) were female, with a mean age at onset of 54.6 ± 15.7 years. No statistically significant differences were observed among the three groups in terms of sex distribution, age at onset, or time from symptom onset to diagnosis. Patients with strongly positive versus weakly positive autoantibody titers exhibited significant differences only in serum levels of CK, lactate dehydrogenase (LDH), aspartate aminotransferase (AST), alanine aminotransferase (ALT), and C-reactive protein (CRP) (**Supplementary Table 1**).

**Table 1:** Characteristics of three subgroups with different IMNM.

Variables	Total N = 133	HMGCR (n=38)	SRP (n=60)	Negative (n=35)	*P* value
Demographic					
Female*, n/N (%)	92/133(69.2%)	31/38(81.6%)	40/60(66.7%)	21/35(60.0%)	0.116
Onset age, years*	54.6(15.7)	54.7(17.7)	54.7(14.7)	54.5(15.4)	0.998
Clinical manifestations					
General					
Fever*, n/N (%)	43/133(32.3%)	11/38(28.9%)	26/60(43.3%)	6/35(17.1%)	0.027
Mucocutaneous					
Rash, n/N (%)	19/133(14.3%)	7/38(18.4%)	10/60(16.7%)	2/35(5.7%)	0.234
Musculoskeletal					
mRS >2	59/133(44.4%)	13/38(34.2%)	26/60(43.3%)	20/35(57.1%)	0.140
Neck weakness*, n/N (%)	51/133(38.3%)	15/38(39.5%)	20/60(33.3%)	16/35(38.3%)	0.481
Muscle weakness*, n/N (%)	116/133(87.2%)	33/38(86.8%)	50/60(83.3%)	33/35(94.3%)	0.303
Myalgia*, n/N (%)	54/133(40.6%)	16/38(42.1%)	21/60(35.0%)	17/35(48.6%)	0.419
Pulmonary					
Dyspnea*, n/N (%)	55/133(41.4%)	10/38(26.3%)	32/60(53.3%)	13/35(37.1%)	0.025
ILD*, n/N (%)	54/133(40.6%)	12/38(31.6%)	34/60(56.7%)	8/35(22.9%)	0.002
RPILD*, n/N (%)	3/133(2.3%)	0/38(0.0%)	2/60(3.3%)	1/35(2.9%)	0.535
Respiratory failure*, n/N (%)	21/133(15.8%)	4/38(10.5%)	15/60(25.0%)	2/35(5.7%)	0.026
Pulmonary hypertension, n/N (%)	3/133(2.3%)	1/38(2.6%)	2/60(3.3%)	0/35(0.0%)	0.563
Cardiovascular					
Cardiovascular disease, n/N (%)	59/133(44.4%)	17/38(44.7%)	31/60(51.7%)	11/35(31.4%)	0.160
Pericardial effusion, n/N (%)	20/133(10.5%)	4/38(15.0%)	9/60(23.3%)	7/35(20.0%)	0.527
Gastrointestinal					
Dysphagia*, n/N (%)	31/133(23.3%)	4/38(10.5%)	14/60(23.3%)	13/35(37.1%)	0.027
Complications					
Pulmonary infection, n/N (%)	61/133(45.9%)	11/38(28.9%)	31/60(51.7%)	19/35(54.3%)	0.045
Bacterial infection, n/N (%)	50/133(37.6%)	9/38(23.7%)	23/60(38.3%)	18/35(51.4%)	0.050
Viral infection, n/N (%)	10/133(7.5%)	2/38(5.3%)	7/60(11.7%)	1/35(2.9%)	0.240
Other CTDs, n/N (%)	23/133(17.3%)	8/38(21.1%)	12/60(20.0%)	3/35(8.6%)	0.280
Laboratory findings					
ANA, n/N (%)	96/133(72.2%)	27/38(71.1%)	49/60(81.7%)	20/35(57.1%)	0.036
Anti-Ro52, n/N (%)	39/133(29.3%)	7/38(18.4%)	29/60(48.3%)	3/35(8.6%)	<0.001
RF positive, n/N (%)	33/133(24.8%)	6/38(15.8%)	20/60(33.3%)	7/35(20.0%)	0.109
Hemoglobin, g/L	131.0(121.0,143.0)	135.0(126.0,143.2)	126.0 (117.0, 143.2)	131.0(124.7,143.2)	0.325
Lymphocytes count, cells/ul	1.62(1.10,2.05)	1.78(1.47,2.37)	1.58(0.91,2.03)	1.54(0.98,1.98)	0.195
Neutrophils count, cells/ul	4.72(3.42,6.87)	5.58(3.95,7.12)	4.05(2.79,6.09)	4.32(3.39,6.93)	0.113
Platelet, cells/ul	241.0(200.5,303.5)	253.0(211.7,320.3)	222.0(187.7,301.5)	237.5(204.7,283.0)	0.582
AST, U/L	100.00(41.50,208.60)	70.75(32.50,188.05)	103.95(31.50,209.65)	100.50(57.30,203.25)	0.844
ALT, U/L	86.30(33.65,150.00)	95.55(33.78,169.28)	78.50(32.00,133.13)	86.65(32.13,171.45)	0.599
CK, U/L*	3063.00(818.00,6435.00)	3755.00(1277.75,6601.00)	2409.00(204.25,6249.00)	3942.00(1524.50,7279.50)	0.262
LDH, U/L*	533.00(233.00,878.50)	408.00(209.75,782.00)	530.50(118.75,1034.00)	493.00(312.25,854.00)	0.544
hypocomplementemia	3/133(2.3%)	1/38(2.6%)	2/60(3.3%)	0/35(0.0%)	0.827
LDL, mmol/L	2.95(2.28,3.48)	2.91(2.48,3.23)	2.75(2.28,3.46)	2.85(2.09,3.32)	0.623
HDL, mmol/L	1.00(0.85,1.30)	1.01(0.91,1.33)	1.01(0.82,1.28)	0.92(0.85,1.29)	0.354
Elevated CRP*, n/N (%)	124/133(93.2%)	34/38(89.5%)	57/60(95.0%)	33/35(94.3%)	0.546
CRP, ng/ml	6.75(3.02,20.15)	6.47(2.92,17.42)	6.66(3.02,27.74)	9.53(3.61,16.22)	0.597
ESR, mm/h	35.0(20.0,59.0)	42.5(25.0,63.7)	38.0(20.0,61.5)	29.0(21.7,70.0)	0.277
IL6**, ng/ml	6.0(3.0,10.0)	5.5(3.0,8.2)	5.5(4.0,10.0)	3.0(6.0,10.0)	0.928
Ferritin***, ng/ml	250.0(153.0,400.0)	216.0 (141.0,330.5)	300.0(200.0,412.5)	250.0(118.2,357.5)	0.131
Imaging finding					
NSIP, n/N (%)	38/133(28.6%)	5/38(13.2%)	21/60(35.0%)	12/35(34.3%)	0.041
OP, n/N (%)	10/133(7.5%)	1/38(2.6%)	5/60(8.3%)	4/35(11.4%)	0.259
NSIP+OP, n/N (%)	46/133(34.6%)	7/38(18.4%)	31/60(51.7%)	8/35(22.9%)	0.001
UIP, n/N (%)	8/133(6.0%)	1/38(2.6%)	5/60(8.3%)	2/35(5.7%)	0.453
Pulmonary function tests					
FVC#, %	86.2(75.0,100.2)	87.0(78.0,98.3)	86.2(72.0,101.5)	81.8(66.1,96.6)	0.498
FEV1##, %	86.4(69.7,95.3)	86.4(76.0,93.9)	87.0(68.9,98.5)	72.3(62.9,86.5)	0.229
FEV1/FVC###	82.0(76.2,86.8)	80.5(74.2,85.0)	84.0(77.5,87.4)	82.0(77.4,86.2)	0.626
DLCO####, %	81.0(68.5,89.3)	82.5(69.3,90.0)	80.1(67.5,88.5)	77.5(61.4,92.0)	0.730
Treatment during this admission					
GC dose>1mg/kg/d	39/133(29.3%)	13/38(34.2%)	16/60(26.7%)	10/35(28.6%)	0.722
Immunosupppressants					
MTX, n/N (%)	60/133(45.1%)	17/38(44.7%)	29/60(48.3%)	14/35(40.0%)	0.716
CTX, n/N (%)	9/133(6.7%)	5/38(13.2%)	3/60(5.0%)	1/35(2.9%)	0.130
CNI, n/N (%)	4/133(3.0%)	2/38(5.3%)	2/60(3.3%)	0/35(0.0%)	0.368
MMF, n/N (%)	28/133(21.1%)	10/38(26.3%)	13/60(21.7%)	5/35(14.3%)	0.411
IVIG, n/N (%)	4/133(3.0%)	3/38(7.9%)	1/60(1.7%)	0/35(0.0%)	0.131
Follow-up duration, month	36.0(15.0,60.0)	26.0(9.7,50.5)	38.0(19.3,60.0)	36.0(12.0,68.0)	0.198
Duration from onset of symptoms to diagnosis, month	5.0(2.0,8.0)	5.5(2.7,12.0)	5.0(2.0,9.7)	4.0(2.0,6.0)	0.333

*Variables used in the unsupervised hierarchical clustering analysis.

Other CTDs include rheumatic arthritis, systemic lupus erythematous, systemic sclerosis and Sjögren’s syndrome.

**IL6 data available for 116 patients.

*** Ferritin data available for 116 patients.

#FVC data available for 74 patients.

##FEV1 data available for 74 patients.

###FEV1/FVC data available for 74 patients.

####DLCO data available for 77 patients.

&lmmunosuppressants included in the treatment were cyclophosphamide, cyclosporine A, tacrolimus, mycophenolate mofetil, azathioprine, methotrexate, leflunomide and thalidomide.

mRS, modified Rankin Scale; scores > 2 indicate moderate to severe disability; ILD, interstitial lung disease; RPILD, rapidly progressive interstitial lung disease; CTDs, connective tissue diseases; ANA, antinuclear antibody; RF, rheumatoid factor; AST, aspartate aminotransferase; ALT, alanine aminotransferase; CK, creatine kinase; LDH, lactate dehydrogenase; LDL, low-density lipoprotein; HDL, high-density lipoprotein; CRP, C reactive protein; ESR, erythrocyte sedimentation rate; IL6, interleukin-6; NSIP, non-specific interstitial pneumonia; OP, organising pneumonia; UIP, usual interstitial pneumonia-like pattern;DLCO, percent predicted diffusing lung capacity for carbon monoxide; FEV1/FVC, forced breathing volume in the 1 s/forced vital capacity; GCs, glucocorticoids; MTX, methotrexate; CTX, cyclophosphamide; CNI, calcineurin inhibitor; MMF, mycophenolate mofetil.

Dyspnea was a common symptom in the cohort (41.4%), with 54 patients (40.6%) developing ILD. Anti-SRP-positive IMNM had a significantly higher ILD incidence (*P* = 0.002) and respiratory failure (*P* = 0.026) than anti-HMGCR-positive IMNM and seronegative IMNM patients. Additionally, fever occurred more frequently in anti-SRP-positive IMNM patients than in the other two subgroups (*P* = 0.027).

With regard to laboratory findings, the frequencies of antinuclear antibody (ANA) and anti-Ro52 antibody positivity were significantly lower in seronegative IMNM patients than in anti-SRP-positive and anti-HMGCR-positive groups (*P* = 0.036 and *P* < 0.001, respectively).

### 3.2 IMNM patients with different MSA presented no differences in prognoses

**Figure 1:**
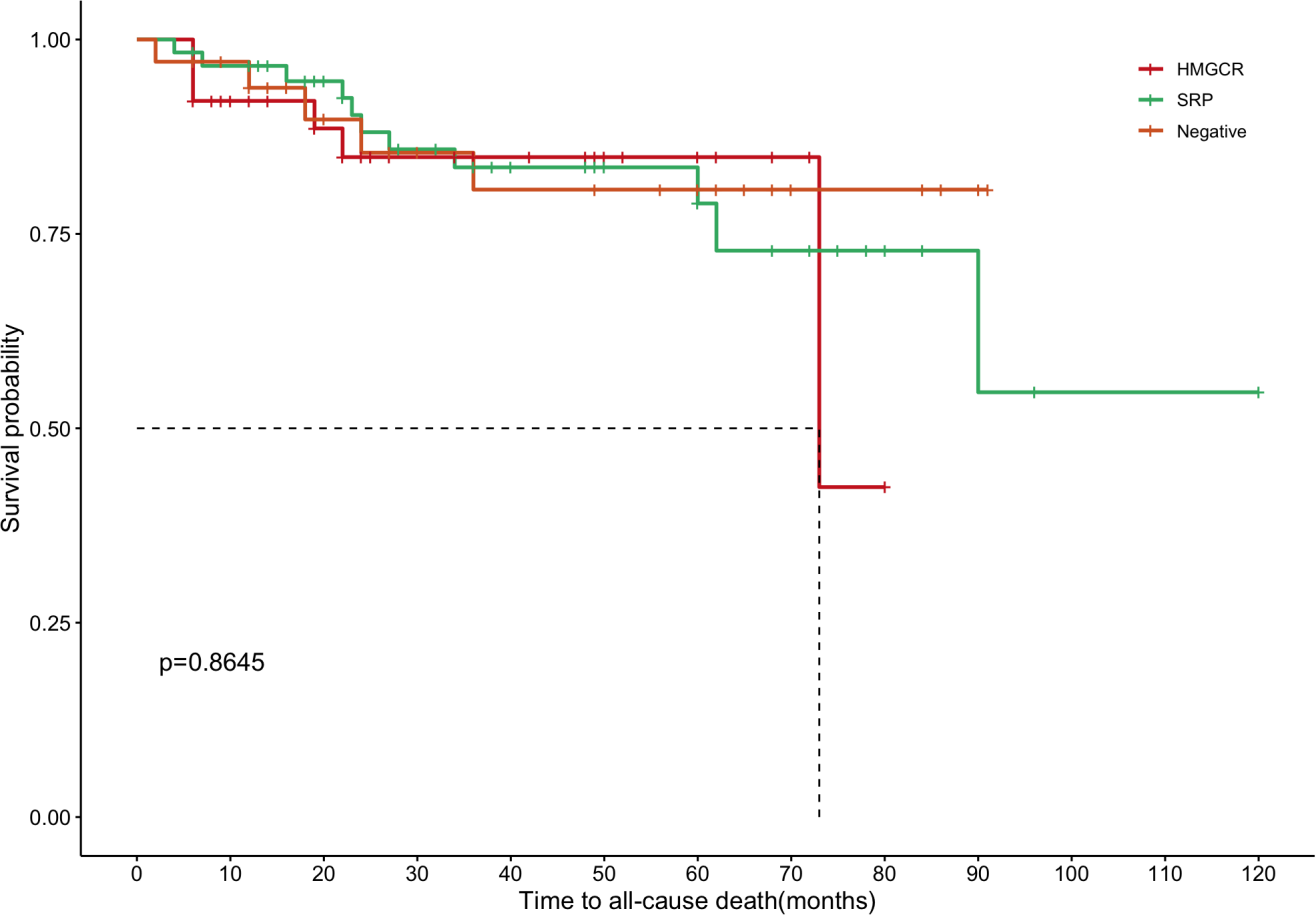
Kaplan–Meier survival curves for immune-mediated necrotizing myopathy patients stratified by myositis specific autoantibody profiles. Survival disparities across antibody subgroups were statistically evaluated via the log-rank test.

We evaluated the outcome of patients according to the MSA classification. The median follow-up duration for this longitudinal cohort was 36.0 months (range, 2.0–120.0 months). There was no significant difference in the three serological subgroups in the median follow-up duration (*P* = 0.198, **Table 1**). Of the 133 patients, 111 (83.5%) were confirmed as survivors, 22 (16.5%) as non-survivors, and 8 (6.1%) were lost to follow-up. Among the 22 non-survivors, the primary causes of death were as follows: 16 patients (72.7%) died of respiratory failure, 3 (13.6%) due to cardiocerebrovascular diseases, 1 (4.5%) died of lung cancer, 1 (4.5%) died of diabetic nephropathy, and 1 (4.5%) died of hepatic failure. Details regarding the distribution of causes of death among the three clusters are presented in **Supplementary Table 2**. Notably, no significant prognostic differences were observed among the three antibody-based groups (*P* = 0.864; **Figure 1**). Specifically, the 1-year survival rates were 92.1% for anti-HMGCR-positive IMNM, 96.6% for anti-SRP-positive IMNM, and 93.7% for seronegative patients. For 5-year survival rates, the corresponding values were 84.8%, 78.9%, and 80.7%, respectively.

### 3.3 Three novel subgroups of IMNM patients with distinct clinical phenotypes were identified, independent of MSA

**Figure 2:**
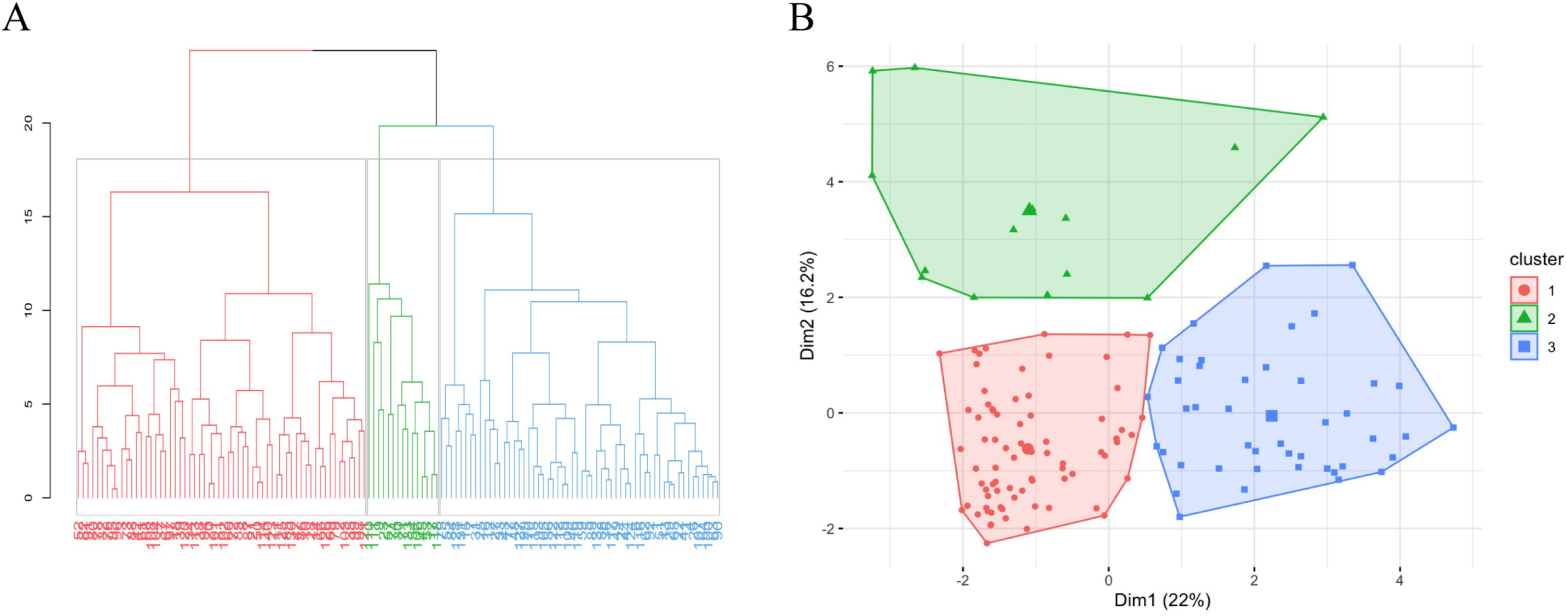
Hierarchical clustering dendrogram and patient stratification in IMNM. **(A)** Unsupervised hierarchical cluster analysis identified three distinct patient subgroups within the IMNM cohort, with classification independent of MSA status. **(B)** Factor distribution plot displaying individual patients, where color coding denotes membership in the three previously defined clusters.

**Figure 3:**
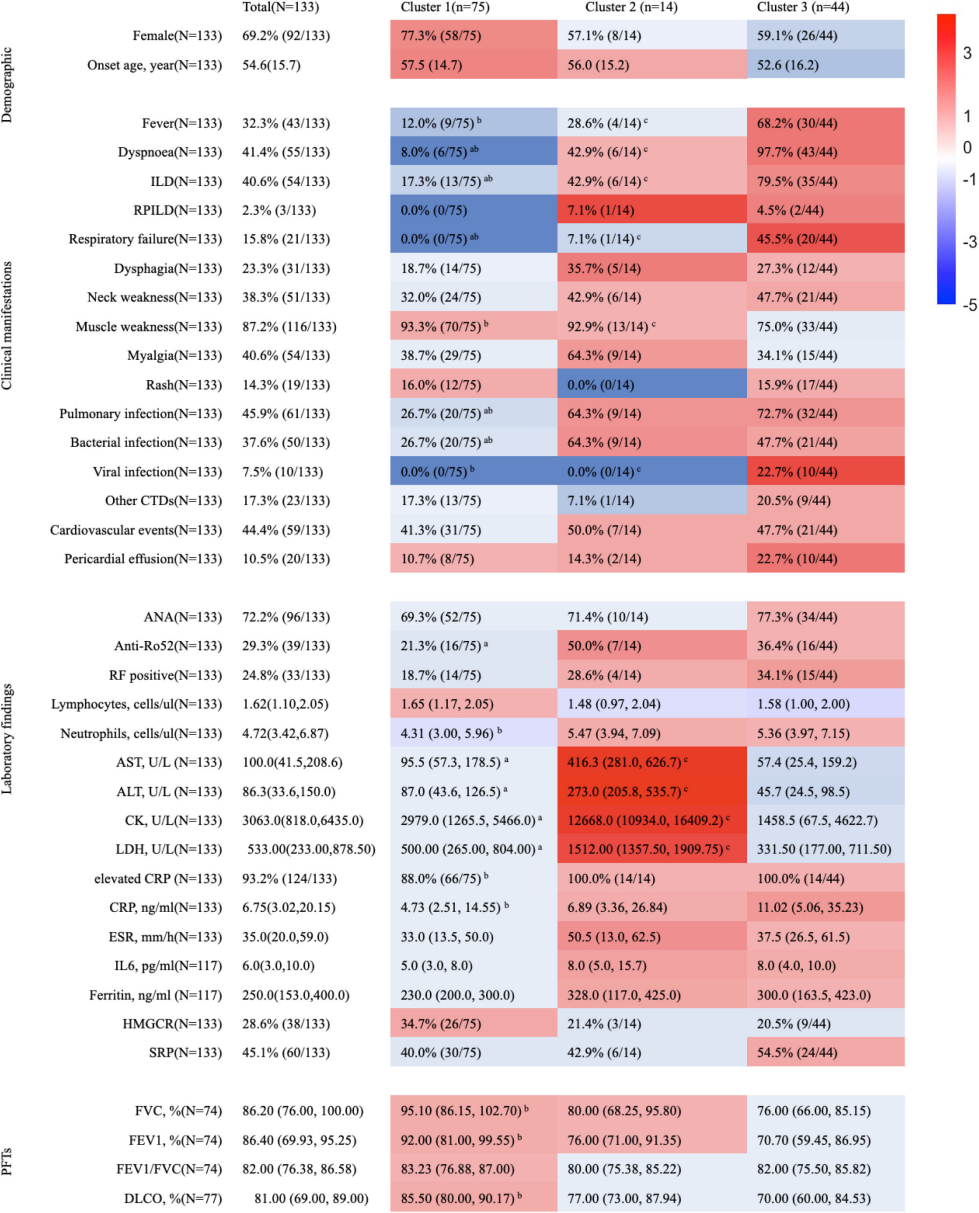
Clinical and laboratory phenotypic features of the three IMNM clusters identified by hierarchical clustering. Continuous variables are reported as either mean ± standard deviation or median (interquartile range, IQR; 25th–75th percentiles), while categorical variables are expressed as counts and corresponding percentages. The color code indicates a score that represents the difference between each subtype and the others (red is positive and blue is negative), which is calculated as the ratio of the value for each cluster to the value for all patients (if resulting value is less than 1, the negative reciprocal is calculated as the score and it is marked as blue). Superscript letters next to cluster labels denote statistically significant differences between subgroups: analysis of variance (ANOVA) or Kruskal-Wallis test for continuous variables, and chi-squared (χ²) or Fisher’s exact test for categorical variables. Specifically, *a* = significant difference between Cluster 1 and Cluster 2; *b* = significant difference between Cluster 1 and Cluster 3; *c* = significant difference between Cluster 2 and Cluster 3. ANA, antinuclear antibody; CCP, cyclic citrullinated peptide; CK, creatine kinase; CRP, C reactive protein; CTDs, connective tissue diseases; DLCO, percent predicted diffusing capacity for carbon monoxide; ESR, erythrocyte sedimentation rate; FEV1/FVC, forced expiratory volume in the 1 second/forced vital capacity; LDH, lactate dehydrogenase; PFTs, pulmonary functional tests; RF, rheumatoid factor; RPILD, rapidly progressive interstitial lung disease.

**Figure 4:**
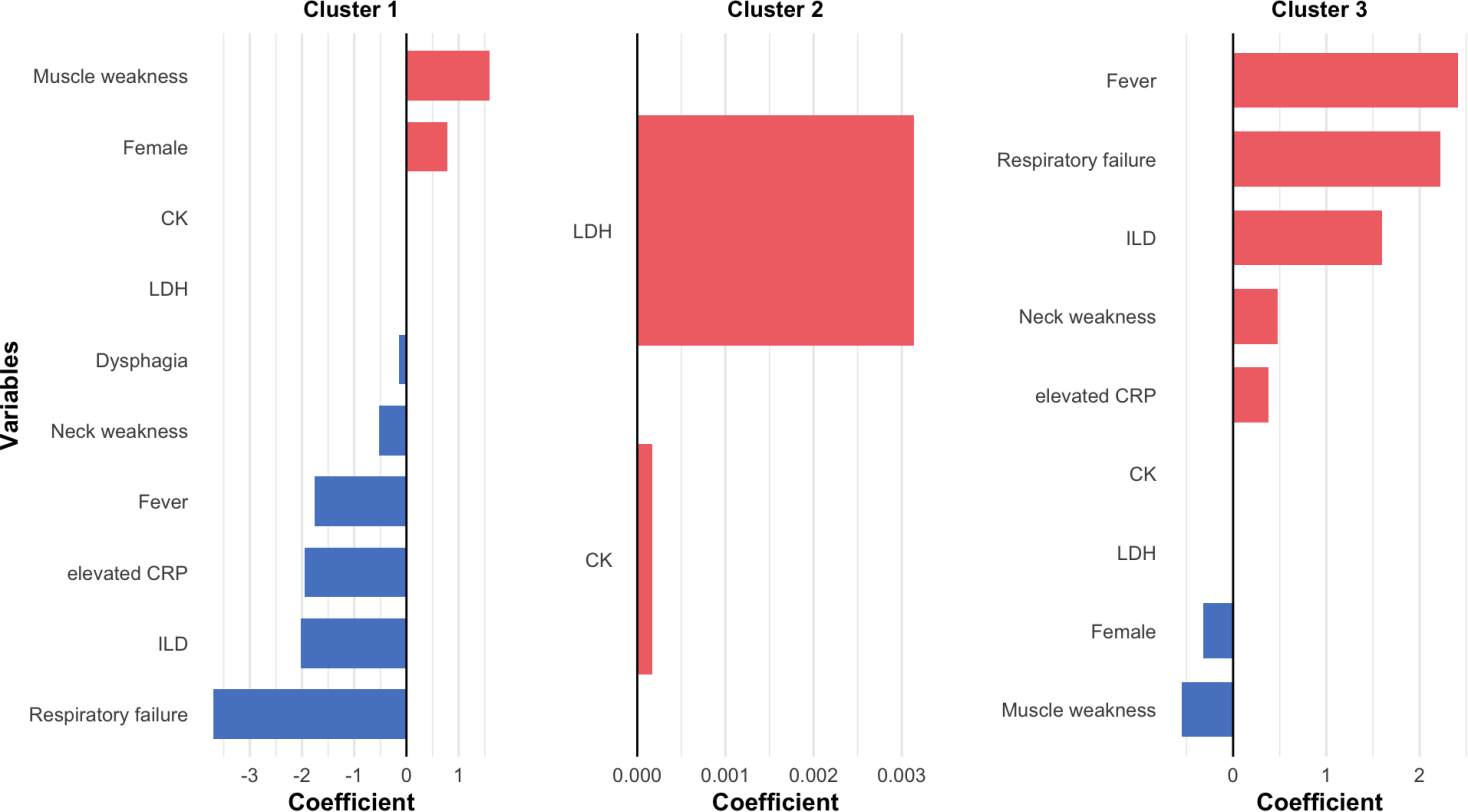
Clinical characteristics with the strongest and weakest associations with each IMNM cluster. Least absolute shrinkage and selection operator regression was employed to identify the most discriminative variables that distinguish each subtype from the others. The y-axis lists the selected clinical variables, with color coding reflecting the direction of their association with the cluster. The x-axis represents the regression coefficients of these variables. CK, creatine kinase; CRP, C reactive protein; LDH, lactate dehydrogenase; RP-ILD, rapidly progressive interstitial lung disease.

Next, we explored whether unsupervised hierarchical clustering could sort IMNM patients into subgroups with more consistent clinical features and prognostic outcomes. For this analysis, we used clinical characteristics and routine lab tests, without considering antibody specificities. We selected fourteen easily obtainable variables that are useful for diagnosing IMNM and assessing disease severity (marked with an asterisk (*) in **Table 1**). As shown in the cluster dendrogram and factor map (**Figures 2A, B**), patients were divided into three distinct phenotypes. Key features of each cluster are presented in **Figure 3**, while the most relevant clinical traits for each group (identified via LASSO regression) are shown in **Figure 4**.

Cluster 1 included 75 patients (56.4% of the total). These patients were mainly characterized by muscle weakness: 93.3% experienced this symptom, a significantly higher rate than in other clusters (*P* = 0.012). In contrast, they had the lowest rates of ILD (17.3%), dyspnea (8.0%), and respiratory failure (0.0%)-all statistically significant differences (*P* < 0.001). LASSO regression results indicated that muscle weakness and female sex were the strongest markers for Cluster 1, while respiratory failure was the least associated trait.

Cluster 2 consisted of 14 patients (10.5%), defined by “high-CK phenotype” features-including severe muscle damage. Muscle weakness was also common (92.9%, *P* < 0.001), and these patients had the highest median levels of several enzymes: CK (12,668.0 U/L), lactate dehydrogenase (LDH, 1,512.0 U/L), aspartate transaminase (AST, 416.35 U/L), and alanine transaminase (ALT, 273.0 U/L). All these differences were confirmed by Kruskal-Wallis H tests (*P* < 0.001).

Cluster 3 included 44 patients (33.1%), characterized by severe pulmonary involvement. Nearly all patients in this Cluster had dyspnea, and they were much more likely to develop ILD (79.5%) and respiratory failure (45.5%) compared to the other two groups (both *P* < 0.001). LASSO regression further verified that respiratory failure and ILD were the key traits for Cluster 3. Additionally, this group had high rates of fever (68.2%, *P* < 0.001) and viral infection (100.0%, *P* < 0.001). They also showed the highest CRP level (11.02 ng/mL, median, Kruskal-Wallis H test, *P* < 0.001) compared with the other two clusters.

### 3.4 IMNM clusters identified independent of antibodies showed different prognoses

**Figure 5:**
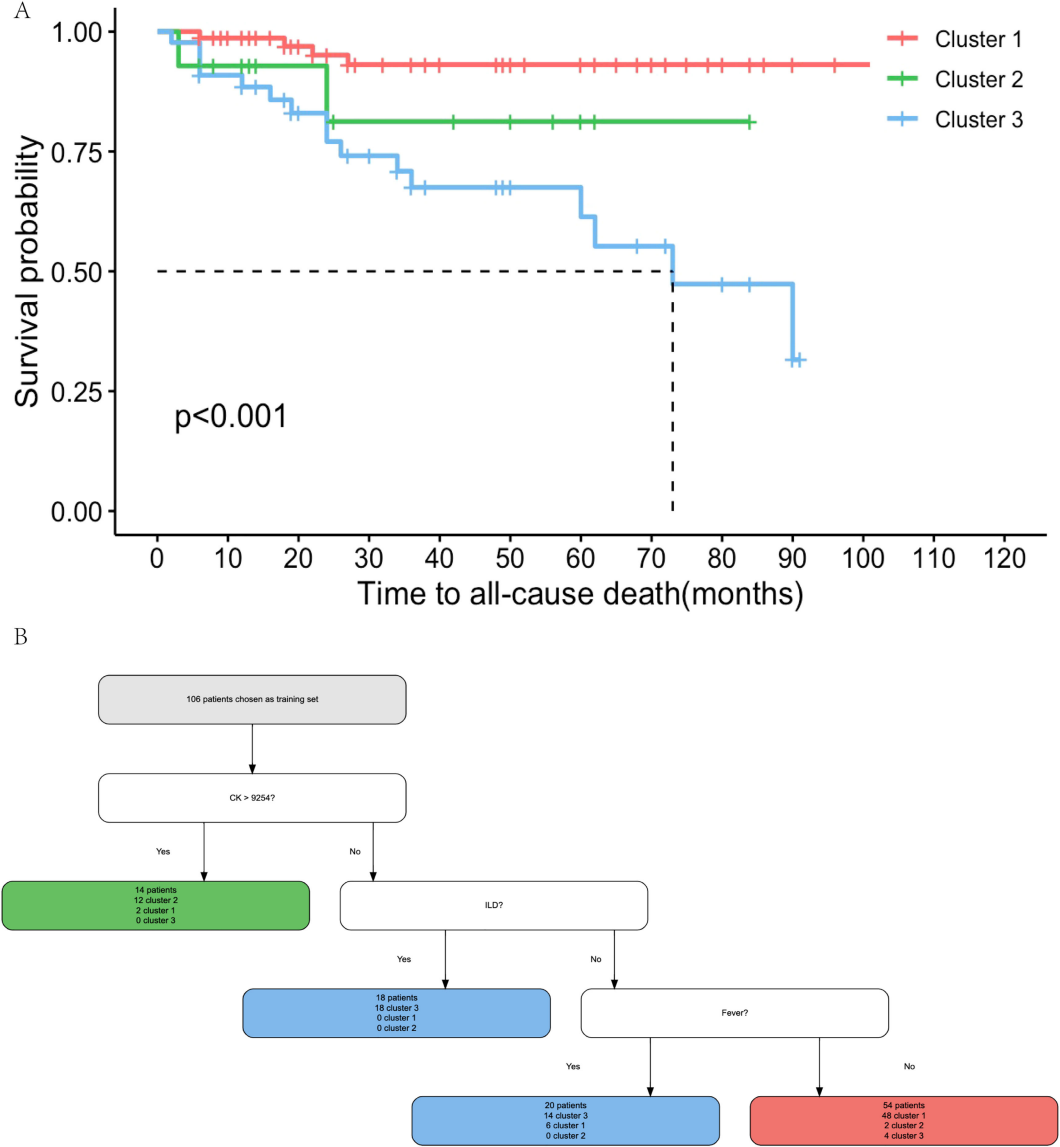
Prognoses outcomes of IMNM patients in different subgroups identified by hierarchical cluster analysis. **(A)** Kaplan-Meier survival curve of patients with different subtypes. The log rank test was performed to compare survival rates. Pairwise comparison showed the survival by cluster differed significantly between Cluster 1 and other clusters. Survival curves showed that Cluster 3 (blue) had a poor prognosis (*P* < 0.001, compared with Cluster 1). **(B)** A pruned prediction model was established by three variables: CK, ILD and fever in the training set. The color coded was pertinent in parts **(A, B)**.

We evaluated the long-term survival of IMNM patients across the three identified clusters. Kaplan–Meier survival analysis confirmed significant prognostic differences among the clusters (*P* < 0.001; **Figure 5A**). Pairwise comparisons further revealed that Cluster 3 had a markedly higher mortality rate than Cluster 1 (*P* < 0.001). The 1-year survival rates of patients in Clusters 1, 2 and 3 were 98.6%, 92.3% and 88.5%, respectively, while the 5-year survival rates were 93.1%, 80.7% and 61.4%, respectively.

### 3.5 Development and validation of the prediction model for IMNM clusters

To facilitate classification of individual patients into the three clusters, we developed a prediction model using the CART algorithm. A total of 133 patients were randomly split into a training set (n = 106) and a validation set (n = 27). RPILD was excluded from the CART analysis due to its strong association with prognosis, leaving 13 variables for model construction. The initial prediction model was derived from the training set and subsequently simplified to include three key variables: CK, ILD, and fever (**Figure 5B**). The model achieved a prediction accuracy of 85.2% in the training set and 84.0% in the validation set.

### 3.6 Proteomic profiling reveals cluster-specific protein signatures

**Figure 6:**
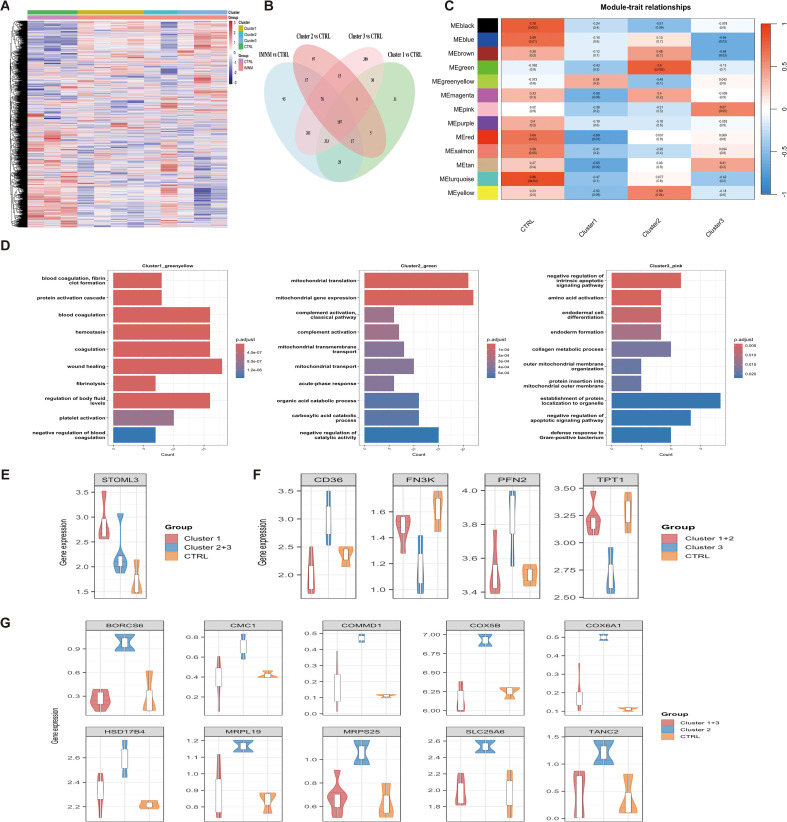
Unique proteomic profiles and associated biological processes of the three IMNM clusters. Muscle tissue proteomic analysis was conducted on 9 IMNM patients (4 from Cluster 1, 2 from Cluster 2, and 3 from Cluster 3) and 3 noninflammatory controls (CTRLs) subjects. **(A)** Heatmap of differentially expressed proteins (DEPs) between the combined IMNM cohort and CTRL. Hierarchical clustering was performed on 9 IMNM samples and 3 CTRL samples based on the expression levels of these DEPs, with color intensity representing row z-scores. **(B)** Venn diagram illustrating the overlap of DEPs identified by comparing CTRLs with each individual IMNM cluster (filter criteria: |log_2_ fold change| ≥ 1.2, *P* < 0.05). **(C)** Module-trait correlation heatmap generated via weighted gene co-expression network analysis, showing the relationship between module eigengenes (MEs) and IMNM sample groups. Each row corresponds to an ME, and each column represents a study group. The correlation coefficient and corresponding *P*-value for each ME–group pair are displayed within the respective cell. The heatmap is color-coded according to the correlation direction and strength (red = positive correlation, blue = negative correlation), as indicated by the color legend. **(D)** Gene Ontology (GO) enrichment analysis of biological processes for proteins within the modules most strongly associated with each IMNM cluster. The top 10 significantly enriched GO biological process terms are shown for each cluster, with color coding indicating the *P*-value of enrichment. **(E)** Violin plots demonstrating the upregulation of key proteins in Cluster 1 relative to CTRL and the other two IMNM clusters. **(F)** Violin plots showing the dysregulation of key proteins in Cluster 3 compared to CTRL and the remaining clusters. **(G)** Violin plots illustrating the upregulation of key proteins in Cluster 2 relative to CTRL and the other two clusters.

To explore potential differences in pathogenic pathways among the three clusters, we performed label-free DIA quantitative proteomic analysis. Muscle samples were collected from 9 IMNM patients (4 from Cluster 1, 2 from Cluster 2, 3 from Cluster 3; detailed clinical information in **Supplementary Table 3**) and 3 noninflammatory controls (CTRLs). Patient selection for proteomic analysis was non-random, with representation of different antibody specificities ensured. Compared with CTRL, IMNM patients exhibited 509 upregulated and 518 downregulated proteins (differentially expressed proteins, DEPs) (**Figure 6A**, **Supplementary Figure 1**). A Venn diagram illustrated the overlap of DEPs between CTRL and each IMNM cluster (**Figure 6B**), indicating cluster-specific DEP expression patterns.

WGCNA was conducted to identify the modules most associated with each cluster. After ensuring the absence of outlier samples (**Supplementary Figure 2A**), an optimal soft thresholding power of 5 was selected (scale-free R^2^ = 0.8590) to construct a scale-free network (**Supplementary Figure 2B**). Dynamic tree cutting was used to identify initial modules (**Supplementary Figure 2C**), which were further merged with a cutHeight of 0.3, resulting in 13 modules (**Figure 6C**). Modules with the strongest eigengene correlations to each cluster were chosen for subsequent analysis. Notably, no module was significantly correlated with Cluster 1 (all *P* > 0.05), although the greenyellow module showed a nominal association (r = 0.39, *P* = 0.201). For Cluster 2 and Cluster 3, the green module (r = 0.8, *P* = 0.002) and pink module (r = 0.57, *P* = 0.05), respectively, showed significant correlations (**Supplementary Figure 3**).

After obtaining the most related module for each cluster, we further investigated their biological significance using GO terms; the top 10 biological processes in the GO analysis of each cluster are shown in **Figure 6D**. Given the exploratory nature of this proteomic analysis and the limited sample size, we performed GO enrichment analysis on the greenyellow module to generate hypothesis−driven biological insights. GO analysis illustrated that proteins in the Cluster 1-related greenyellow module were significantly enriched in blood coagulation, platelet activation, platelet aggregation and protein activation cascade signaling pathways. Proteins in the Cluster 2-related green module were significantly enriched in pathways including mitochondrial translation, complement activation, mitochondrial transmembrane transport, mitochondrial transport and acute-phase response signaling. Furthermore, proteins in the Cluster 3-related pink module were associated with collagen metabolic process and negative regulation of intrinsic apoptotic signaling pathway. We termed the three clusters as different phenotypes, given that the different clusters had specific DEPs, modules of interest and enriched pathways. Different proteins were identified as overlapping proteins for each phenotype (**Supplementary Figure 4**), with 1 protein for phenotype 1, 10 proteins for phenotype 2 and 4 proteins for phenotype 3. Specifically, TANC2, COX5B, BORCS6, SLC25A6, MRPS25, COX6A1, HSD17B4, COMMD1, MRPL19, and CMC1 were identified as key proteins for phenotype 2; and CD36, TPT1, FN3K, and PFN2 were identified as key proteins for phenotype 3. The expression levels of these key proteins are shown in **Figures 6E–G**.

## 4 Discussion

**Figure 7:**
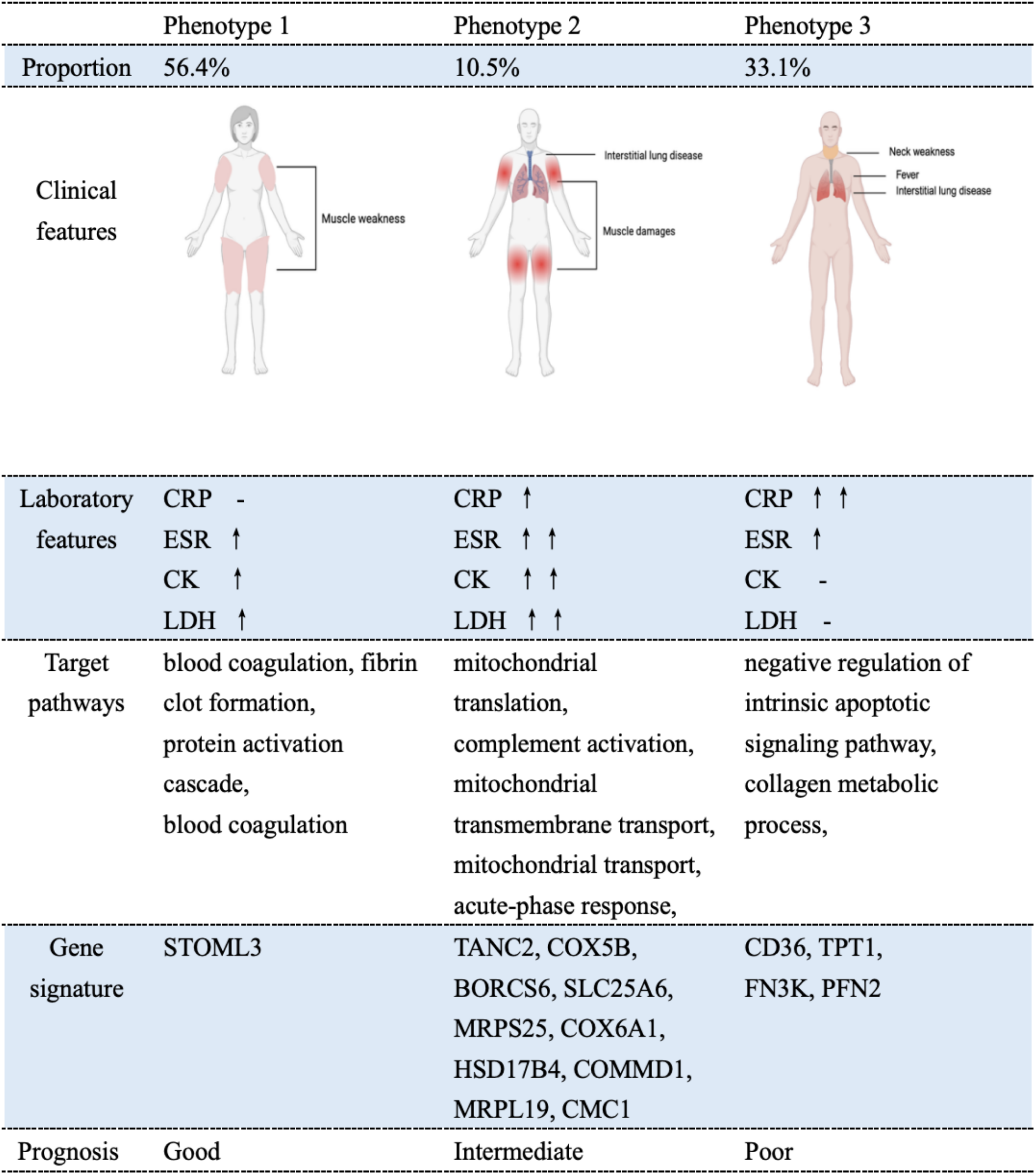
Schematic summary of the core features of the three IMNM phenotypes. This diagram integrates and visualizes the distinct clinical phenotypes, laboratory findings, proteomic signatures, and prognostic outcomes associated with each phenotype. CK, creatine kinase; CRP, C-reactive protein; ESR, erythrocyte sedimentation rate; LDH, lactate dehydrogenase.

In this retrospective cohort study, we found that IMNM patients with different antibodies had a comparable prognosis. We then used unsupervised clustering analysis based on clinical features and laboratory results (not antibody specificity) to classify IMNM patients into three new phenotypes. These phenotypes grouped patients into more homogeneous subgroups, which differed in clinical features, clinical outcome, and protein expression patterns (**Figure 7**).

Previous studies have established an inverse correlation between anti-SRP/anti-HMGCR antibody titers and muscle strength in IMNM patients (23, 24). Beyond this direct association, the observed link between autoantibody titers and CK levels provides support for the pathogenic roles of these autoantibodies in IMNM pathogenesis (25). Building on these findings, our study further confirmed that although distinct antibodies correlate with the degree of muscle damage, none of these serological markers show a statistically significant association with overall survival in IMNM patients. In a related analysis, Yang et al. stratified IMNM patients by MSA status to evaluate treatment responses and clinical outcomes (16). Their assessment of both short-term and long-term follow-up data revealed no significant differences in time to remission or relapse-free survival curves among the three serological subgroups. A recent cohort study conducted in China used log-rank tests and reported that patients positive for anti-SRP antibodies had poorer survival rates compared to those with anti-HMGCR antibodies. Nevertheless, results from multivariate Cox regression analysis revealed that respiratory failure, rather than MSA specificity, was the pivotal prognostic indicator (26). These combined results suggest that when confounding variables are controlled for, mortality in IMNM cohorts is more closely linked to the presence of severe clinical manifestations than to the specific type of MSA. Additionally, our data showed no significant differences in the time interval from symptom onset to definitive diagnosis across the three serological subgroups. This finding helps to further clarify why IMNM patients with varying MSA specificities have comparable prognostic prognoses.

Given the limited value of autoantibodies in predicting clinical outcomes, we adopted an unsupervised clustering approach to stratify IMNM patients into more homogeneous subgroups. Rather than relying on MSA specificity, this classification was based solely on patients’ clinical manifestations and laboratory test results. Three distinct phenotypes were identified, each corresponding to a clinically meaningful subset of IMNM with unique prognostic characteristics.

The present study identified a relatively high prevalence of ILD among IMNM patients, particularly in phenotype 3, which aligns with the findings of prior investigations (3, 5, 27, 28). This high frequency of ILD in our cohort may be due to the inclusion of a subset of patients who initially presented to the respiratory department with predominant dyspnea, while exhibiting only mild or subtle muscle weakness. Although IMNM-related ILD has traditionally been regarded as mild to moderate in severity (7), rare occurrences of acute respiratory failure and RPILD can still increase mortality in IMNM patients (29). Additionally, ILD is widely recognized as a factor linked to poor prognosis in IMNM (5, 7). A retrospective study on IMNM found that ILD was strongly associated with treatment-refractory cases, with an odds ratio of 39.70 (30). Therefore, the identification of phenotype 3 holds considerable clinical value. Given that a subset of IMNM patients initially present to respiratory departments with dominant respiratory symptoms rather than muscle weakness, enhancing awareness of this subtype across multiple clinical specialties (e.g., pulmonology, neurology, and rheumatology) is crucial. Early recognition of this subtype and the implementation of tailored treatment strategies are critical for improving patient survival. Notably, prior studies have pinpointed female sex as a protective factor against ILD development and poor clinical outcomes (3, 27, 31). This finding aligns well with our own data, which showed a lower incidence of ILD in patients classified as phenotype 1.

Phenotypes 1 and 2 constituted the majority of the study cohort (66.9%), both presenting with classic IMNM features, clinically manifested as muscle weakness and elevated CK levels. While phenotype 2 was associated with relatively more severe muscle involvement, no significant difference in prognostic outcomes was observed between phenotype 1 and phenotype 2. These results suggest that the severity of muscle weakness and degree of muscle damage are not prognostic determinants. Interestingly, we noted that the survival curves of phenotypes 1 and 2 were similar initially. However, the curve for phenotype 2 dropped sharply after the 20–month mark, resulting in a clear separation between the two curves from that point onward. A key distinction between these two subgroups was found in the incidence of ILD. Specifically, among the five phenotype 2 cases, ILD developed 12 months after the initial diagnosis of IMNM. We speculate that this survival curve divergence may be related to the higher prevalence of cardiorespiratory dysfunction in phenotype 2 patients. These individuals presented with a lower baseline percentage of predicted diffusing capacity of the lung for carbon monoxide (DLCO) compared with their phenotype 1 counterparts. Given the divergent long-term prognostic outcomes of these two phenotypes, beyond the standard initial assessment and therapy, continuous long-term follow-up and tailored management strategies are vital for this patient population.

Beyond their clear clinical relevance, our findings-which uncovered unique protein expression patterns and core biological pathways across different IMNM phenotypes, also shed new light on the disease pathogenesis. Phenotypes refer to distinct disease subtypes that are characterized by specific intrinsic pathophysiological mechanisms and may respond differently to various therapeutic approaches. At present, there are no standardized treatment protocols for IMNM. For this reason, it is critical to identify disease-specific subtypes and their underlying molecular mechanisms. It is equally important to discover related biomarker signatures, as these can guide clinicians in selecting the most appropriate treatment plans for individual patients.

GO analysis of phenotype 1 identified blood coagulation and platelet activation as the primary enriched pathways, which require further investigation. For phenotype 2, the enriched pathways mainly focused on the mitochondrial translation, complement activation, mitochondrial transmembrane transport, mitochondrial transport and acute-phase response signaling pathways. Previous studies have shown that mitochondria dysfunction and metabolic reprogramming as drivers of idiopathic pulmonary fibrosis (32). Our study confirmed that complement activation exists in skeletal muscle tissue during the process of muscle damage in IMNM, which is consistent with a previous study (33). In addition, Shun et al. reported that elevated serum complement levels were linked to more favorable prognoses in patients with myositis-associated ILD (34). Mitochondrial pathway enrichment suggests bioenergetic impairment, supporting the exploration of mitochondrial protectants (e.g., coenzyme Q10), while the robust acute−phase response indicates heightened systemic inflammation, which could warrant intensified immunosuppression (e.g., rituximab or combination regimens).

For phenotype 3, GO enrichment analysis highlighted two main biological processes: collagen metabolic pathways and the negative regulation of intrinsic apoptotic signaling. Recent multiomics research has shown that IMNM is defined by a distinct molecular profile, with notable enrichment in extracellular matrix (ECM)-related signaling pathways (35). Pulmonary fibrosis, the end-stage pathological manifestation of ILD, is characterized by abnormal fibroblast overgrowth and extensive ECM accumulation (36). This pathological feature helps explain the clinical finding that ILD occurs in both phenotypes 2 and 3, while phenotype 3 is associated with far more severe ILD and a significantly poorer prognosis. Regarding key proteins, thrombospondin-1 (TSP-1) binds to CD36 receptor, which boosts the expression of the pro-apoptotic factor caspase-3; this process then inhibits angiogenesis. Previous studies have reported elevated TSP-1/CD36 binding activity in inclusion body myositis and pulmonary fibrosis (37, 38). In pulmonary fibrosis, myofibroblasts drive ECM deposition and resist apoptosis, promoting disease progression (39). Similarly, muscle fibrosis involves activated myofibroblasts and dysregulated ECM turnover. Given that phenotype 3 showed enrichment of collagen metabolic pathways and negative regulation of intrinsic apoptosis, both hallmarks of myofibroblast persistence, these observations support the notion that similar effector cells and pro−fibrotic factors are dysregulated in both lung and muscle compartments. Importantly, the unique association of phenotype 3 with collagen metabolic processes and negative regulation of intrinsic apoptosis underscores fibrotic remodeling as a dominant pathogenic driver, mediating irreversible muscle damage and refractoriness to conventional immunosuppression. Given phenotype 3’s link to severe ILD, antifibrotic treatments (e.g., nintedanib, pirfenidone) could potentially be effective in patients with refractory myositis−associated ILD, but clinical trials are needed to guide treatment decisions (40). Together, these findings offer deeper insights into the underlying mechanisms driving phenotype 3.

Our study has several key strengths, including a longitudinal patient cohort and the identification of potential links between clinical phenotypes and protein expression profiles. Nevertheless, certain limitations should be acknowledged. First, the retrospective nature of the design inherently introduces unavoidable biases (e.g., confounding factors, incomplete data documentation), and the single tertiary medical center recruitment may lead to selection bias, limiting the generalizability of our findings. While internal validation was conducted, external confirmation through multicenter collaborative studies is imperative. Second, the retrospective design, combined with the small number of patients with severe dysphagia requiring gastric tube intubation and incomplete data on dysphagia severity and gastric tube use, precluded a systematic evaluation of the prognostic impact of this condition. Third, the relatively small proteomic sub cohort (n=9) resulted in non-significant module-trait correlation for Cluster 1 (rendering its GO enrichment results preliminary) and moderate correlations for other modules, highlighting the need for validation in larger cohorts and additional experimental studies.

By applying unsupervised clustering to comprehensive clinical characteristics and routine laboratory indicators, we successfully stratified IMNM patients into three distinct phenotypes. These subgroups displayed notable differences in clinical outcomes and unique protein signature patterns. This novel classification provides fresh insights into the inherent heterogeneity and complexity of IMNM, and may offer valuable guidance for the development of personalized treatment approaches.

## Data Availability

The mass spectrometry proteomics data have been deposited in the iProX database (https://www.iprox.cn) under the accession number PXD078154.
