# Supplementary material for: Novel phenotypes of immune-mediated necrotizing myopathy identified independent of myositis-specific antibody specificity that improve prognostic stratification
Source: Front Immunol. 2026 May 14;17:1821033. doi: 10.3389/fimmu.2026.1821033 (PMC13216009; doi:10.3389/fimmu.2026.1821033)
Supplement: Supplementary file 1 [file DataSheet1.docx]

**1.SUPPLEMENTARY METHODS**

**1.1Protein extraction and mass spectrometry (MS)**

Control muscle samples were obtained from individuals who underwent diagnostic muscle biopsy for clinical suspicion of myopathy (e.g., unexplained weakness). After thorough clinical, laboratory, and histopathological assessment, all controls were confirmed to have normal muscle histology and no evidence of inflammatory myopathy, muscular dystrophy, or other primary muscle disorders. Muscle tissue proteins were extracted using the following protocol: first, muscle samples were cryogenically ground in liquid nitrogen (1-minute pulses, repeated 4 times); next, samples were lysed in SDS buffer for 30 minutes and sonicated on ice at 4 °C (8-second cycles, 10 minutes total); finally, the mixture was centrifuged at 12,000 × g for 20 minutes to collect the supernatant. Protein concentration was quantified using a MicroBCA kit (Thermo Fisher Scientific). Only samples meeting the following criteria were selected for further analysis: protein concentration ≥ 2 μg/μL and total protein yield > 120 μg. As described previously(1), peptides were prepared via acetone precipitation, urea denaturation, and trypsin digestion. Peptide analysis was performed using a data-independent acquisition (DIA) approach coupled with a NanoAcquity UHPLC system and an Orbitrap Exploris 240 mass spectrometer (Thermo Fisher Scientific). A protein library was constructed using human protein sequences retrieved from UniProt (www.uniprot.org/, accessed October 20, 2024), with an FDR threshold of < 0.05.

**1.2 Mass spectrometry (MS) data analysis,** **weighted gene co-expression network analysis, functional analysis, and key gene identification**

The raw files were processed using DIA-NN v1.8.1 and were analyzed by DEP package v1.28.0 (2). This analysis pipeline included normalization, data transformation, and differential expression analysis; the Benjamini-Hochberg procedure was applied to adjust *P*-values. Proteins were excluded if more than 50% of samples in any designated group had missing values.

The “WGCNA” package (version 1.73.1) was utilized for constructing the co-expression network (3). First, samples were clustered using the `hclust` function to identify obvious outliers. Next, an automatic network construction tool was used to establish a scale-free co-expression network, with the R function `pickSoftThreshold` utilized to calculate the optimal soft thresholding power. Subsequently, the adjacency matrix was converted into a topological overlap matrix via hierarchical clustering, and the dynamic tree cut algorithm was applied to define distinct modules. Finally, Pearson correlation coefficients between module eigengenes and different IMNM patient clusters were evaluated.

The ClusterProfiler package (v4.14.6) was used for Gene Ontology (GO) biological process annotation of genes from modules of interest (4). All candidate genes were mapped to terms in the GO database, and the number of genes assigned to each term was counted. A hypergeometric test was performed to identify GO terms significantly enriched in candidate genes compared to the species-specific background gene set. The Benjamini-Hochberg method was used to adjust *P*-values, with a threshold of adjust *P*-value < 0.05 defining significantly enriched GO terms.

Module core genes overlapping with differentially expressed proteins (DEPs) were selected as key protein candidates. Venn diagrams illustrating these significant genes were generated using the “venn” package (v1.12). For descriptive statistics and data visualization, gene counts were normalized (DESeq2 size factor normalization) and transformed (variance stabilization transformation, VST).

**1.3 Unsupervised analysis**

Step 1: The factor analysis of mixed data algorithm was utilized to assess similarity among individuals by incorporating mixed variable types (quantitative and qualitative). During analysis, all variables were normalized to balance the relative influence of each variable set. A factor map was generated to visualize associations between variables and among individuals, with the first two axes prioritized to maximize the explanation of data variance.

Step 2: A hierarchical clustering on principal components approach was applied to identify clusters of samples with similar characteristics. A hierarchical dendrogram was constructed using Ward’s linkage criterion, and samples were ultimately partitioned into three distinct clusters via hierarchical clustering.

**1.4 Regression analysis**

Least absolute shrinkage and selection operator (LASSO) regression was performed to identify the most relevant features for each cluster. The “lambda.1se” parameter was selected based on 10-fold cross-validation, which minimized the prediction error rate while producing a parsimonious model. The most significant variables were presented with their corresponding coefficients and colored according to the direction of their association with each cluster.

**1.5 Classification and Regression Tree (CART) Algorithm**

The CART algorithm was employed to assign each participant to the newly defined subgroups. A total of 133 patients were randomly split into a training set (n = 106) and a validation set (n = 27). The optimal decision tree was selected by balancing the minimum cross-validated prediction error with the trade-off between sensitivity and specificity. A pruned decision tree (minimum cross-validation error: x-error = 0.303; number of splits = 3; complexity parameter = 0.01) was developed, with the following variables incorporated: creatine kinase, ILD, and fever.

**2. SUPPLEMENTAL FIGURES**

**
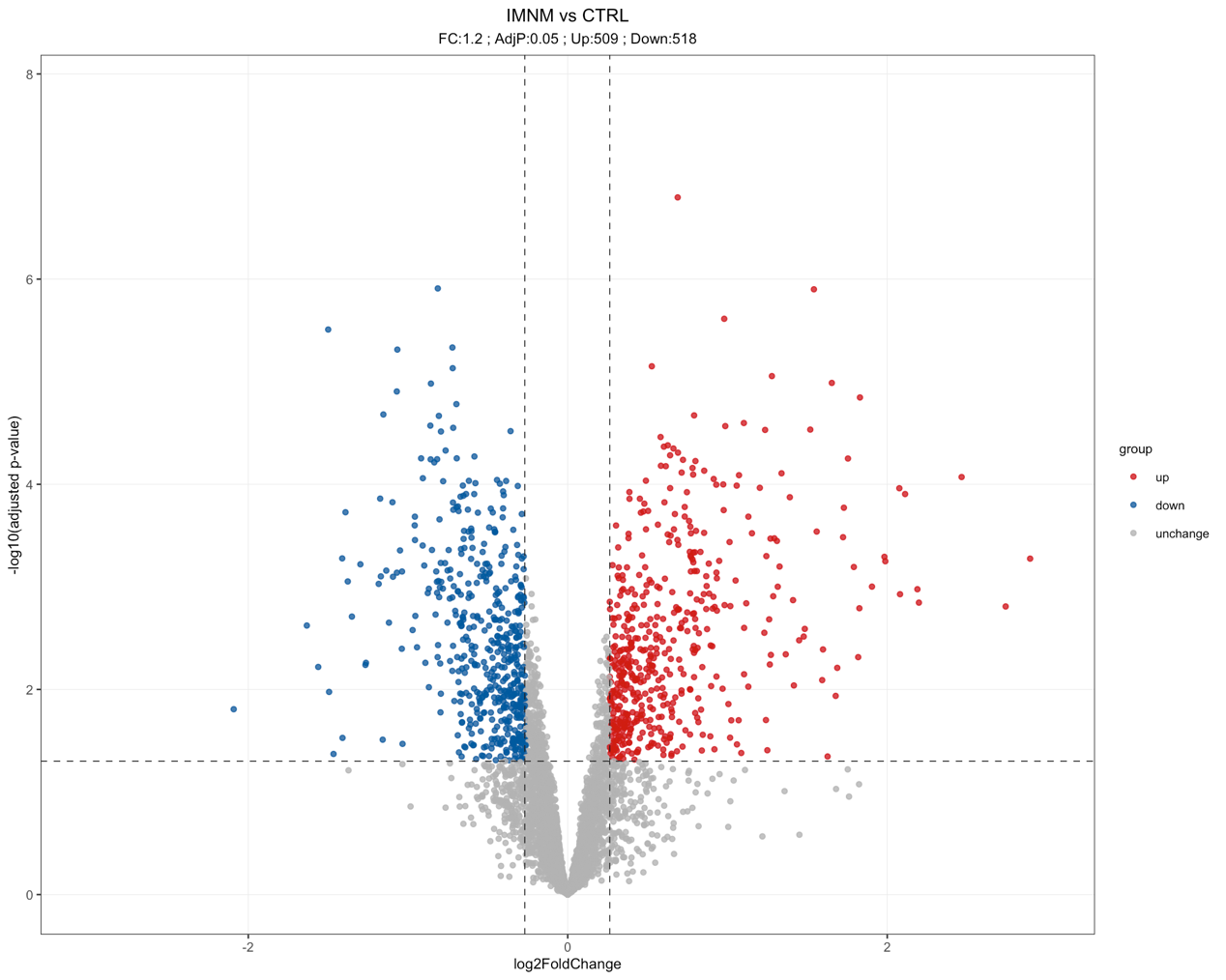
**

**Supplemental Figure S1 Identification of differentially expressed proteins (DEPs) in patients with IMNM.** Volcano plot displaying 1027 DEPs between the IMNM and CTRL groups (｜log2 fold change｜≥1.2, *P*<0.05). Abbreviations: IMNM, immune-mediated necrotizing myopathy; CTRL, noninflammatory control.


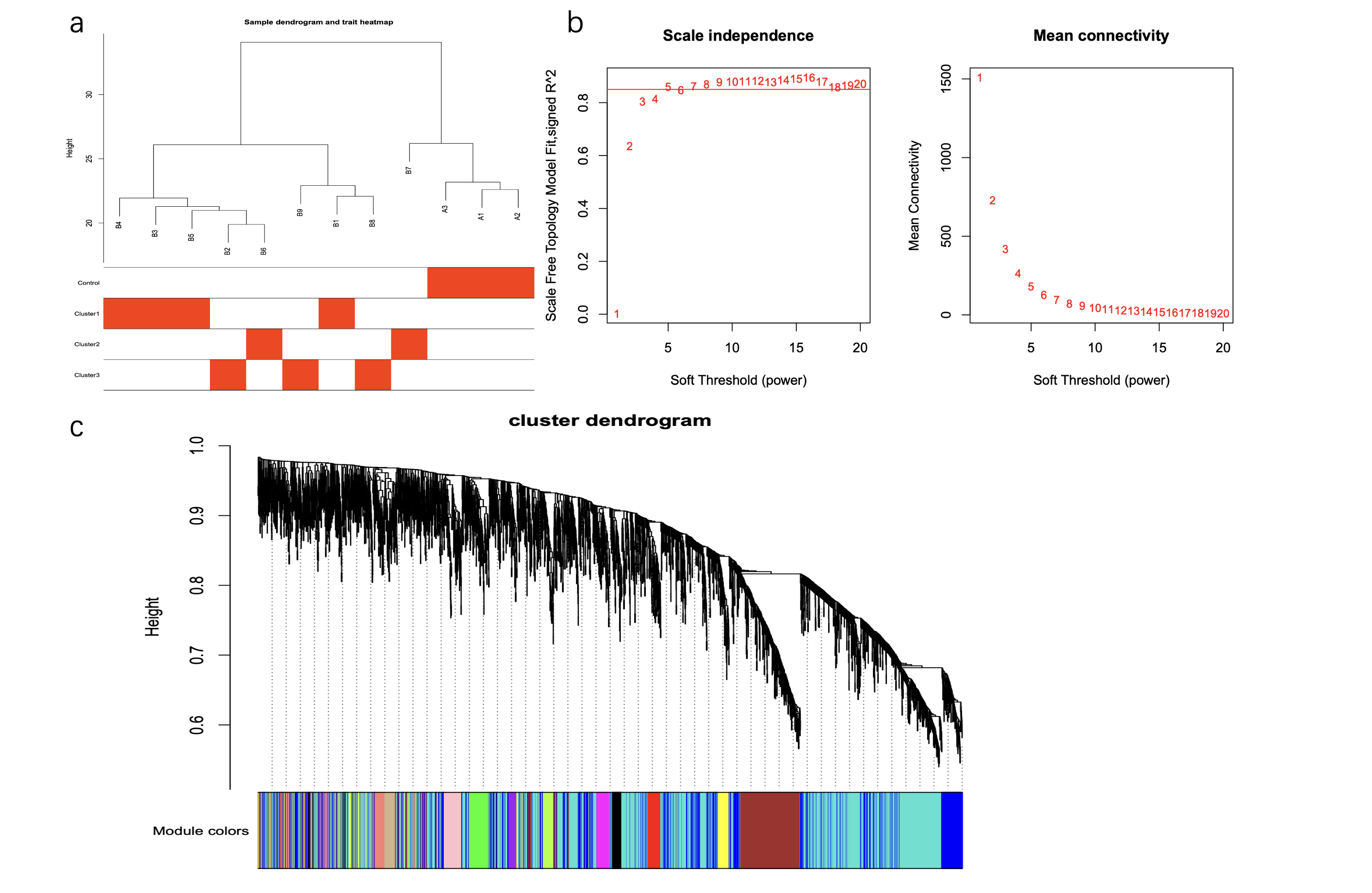


**Supplemental Figure S2 Weighted gene co-expression network analysis.**

a. Sample clustering to detect outliers. All samples were included, and no outlier were identified. b. Analysis of the network topology for selecting the optimal soft threshold power. c. Dendrogram of gene topological overlap matrix. The parameters minimum cluster size and deepSplit were set as 50 and 2, respectively, to construct the primary modules.


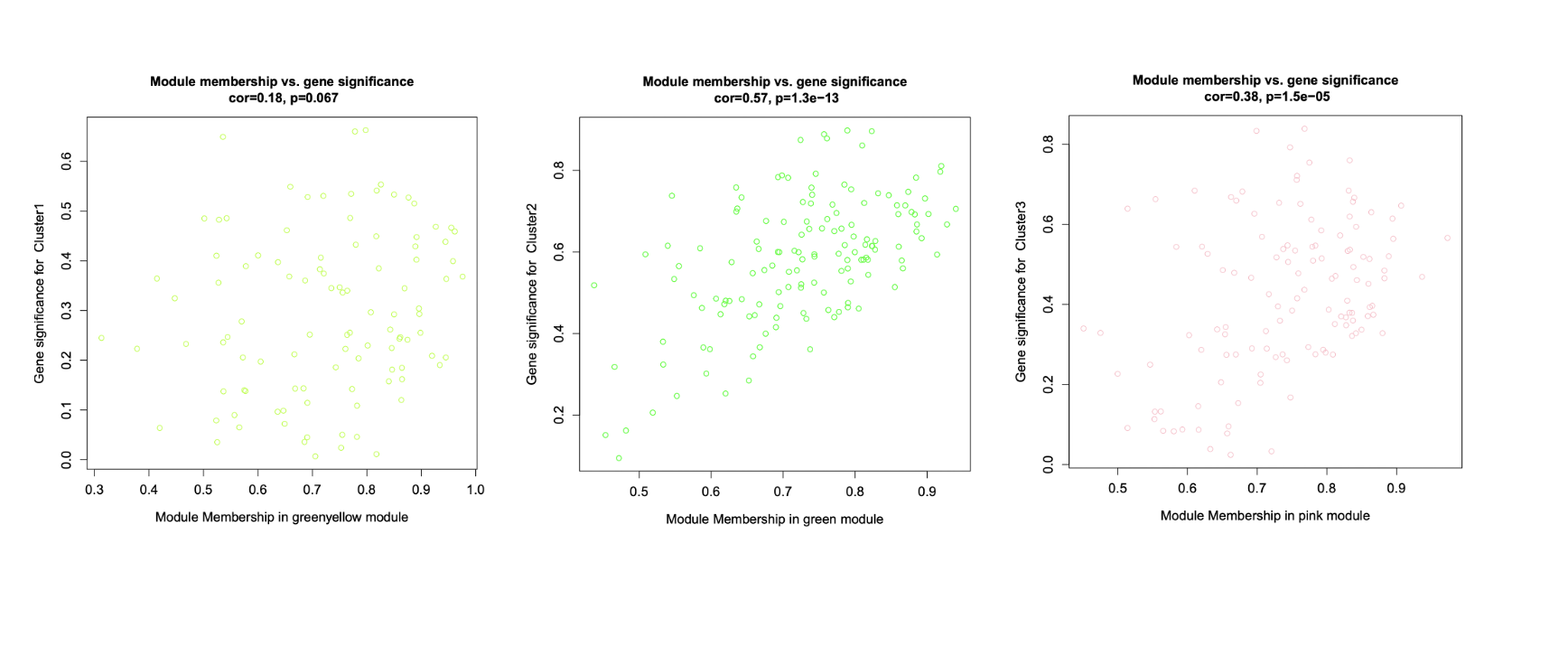


**Supplemental Figure S3 The correlation between module membership and clusters.** Scatter plots of gene significance for different groups vs. module membership in greenyellow, green and pink modules.


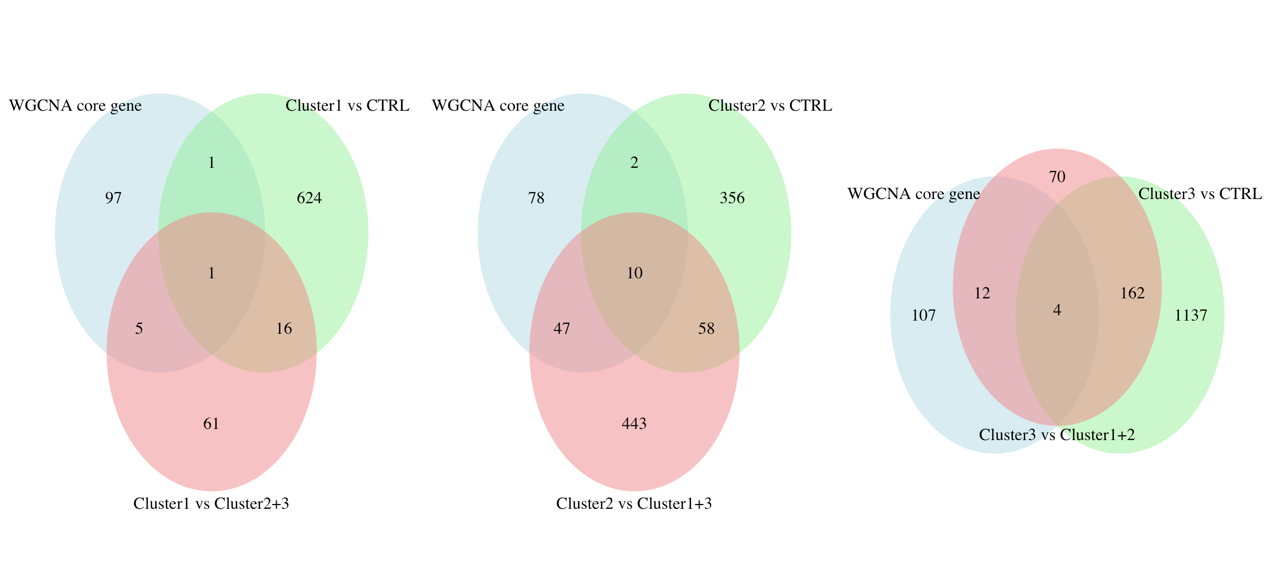


**Supplemental Figure S4 Identification of overlapped genes corresponding to differentially expressed proteins of each phenotype.** Venn diagram revealing overlapped genes of each phenotype. One gene was identified for phenotype 1, ten genes were determined for phenotype 2, and four genes for phenotype 3.

**3. SUPPLEMENTAL TABLE**

**Table S1. Characteristics of IMNM subgroups with strong and weaker signal intensities of antibody.**

| Variables | Total  (N=98) | Strong signal intensity  (n=34) | Weaker signal intensity  (n=64) | *P*-value |
| --- | --- | --- | --- | --- |
| **Demographic data** |  |  |  |  |
| Female, n/N (%) | 71/98(72.4%) | 25/34(73.5%) | 46/64(71.9%) | 0.861 |
| Onset age, years | 54.6(15.7) | 57.7(16.4) | 53.1(15.5) | 0.174 |
| **Clinical manifestations** |  |  |  |  |
| **General** |  |  |  |  |
| Fever, n/N (%) | 37/98(37.8%) | 11/34(32.4%) | 26/64(40.6%) | 0.421 |
| **Mucocutaneous** |  |  |  |  |
| Rash | 17/98(17.3%) | 5/34(14.7%) | 12/64(18.8%) | 0.615 |
| **Musculoskeletal** |  |  |  |  |
| mRS >2 | 39/98(39.8%) | 17/34(50.0%) | 22/64(34.4%) | 0.133 |
| Neck weakness，n/N (%) | 35/98(35.7%) | 14/34(41.2%) | 21/64(32.8%) | 0.411 |
| Muscle weakness, n/N (%) | 83/98(84.7%) | 31/34(91.2%) | 52/64(81.3%) | 0.194 |
| Myalgia，n/N (%) | 37/98(37.8%) | 11/34(32.4%) | 26/64(40.6%) | 0.421 |
| **Pulmonary** |  |  |  |  |
| Dyspnoea, n/N (%) | 42/98(42.9%) | 14/34(41.2%) | 28/64(43.8%) | 0.806 |
| ILD, n/N (%) | 46/98(46.9%) | 15/34(44.1%) | 31/64(48.4%) | 0.683 |
| RPILD, n/N (%) | 2/98(2.0%) | 1/34(2.9%) | 1/64(1.6%) | 0.646 |
| Respiratory failure, n/N (%) | 19/98(19.4%) | 4/34(11.8%) | 15/64(23.4%) | 0.164 |
| Pulmonary hypertension, n/N (%) | 3/98(3.1%) | 1/34(2.9%) | 2/64(3.1%) | 0.960 |
| **Cardiovascular** |  |  |  |  |
| Cardiovascular events, n/N (%) | 48/98(49.0%) | 19/34(55.9%) | 29/64(45.3%) | 0.319 |
| Pericardial effusion, n/N (%) | 13/98(13.3%) | 7/34(20.6%) | 6/64(9.4%) | 0.119 |
| **Gastrointestinal** |  |  |  |  |
| Dysphagia, n/N (%) | 18/98(18.4%) | 10/34(29.4%) | 8/64(12.5%) | 0.040 |
| **Complications** |  |  |  |  |
| Pulmonary infection, n/N (%) | 42/98(42.9%) | 15/34(44.1%) | 27/64(42.2%) | 0.854 |
| Bacterial infection, n/N (%) | 32/98(32.7%) | 12/34(35.3%) | 20/64(31.3%) | 0.684 |
| Viral infection, n/N (%) | 9/98(9.2%) | 3/34(8.8%) | 6/64(9.4%) | 0.928 |
| Other CTDs^a^, n/N (%) | 20/98(20.4%) | 6/34(17.6%) | 14/64(21.9%) | 0.621 |
| **Laboratory findings** |  |  |  |  |
| ANA, n/N (%) | 76/98(77.6%) | 28/34(82.4%) | 48/64(75.0%) | 0.406 |
| Anti-Ro52, n/N (%) | 36/98(36.7%) | 19/34(55.9%) | 17/64(26.6%) | 0.004 |
| RF positive, n/N (%) | 26/98(26.5%) | 10/34(29.4%) | 16/64(25.0%) | 0.638 |
| Lymphocytes count, cells/ul | 1.68(1.13,2.06) | 1.63(1.03,1.99) | 1.71(1.37,2.17) | 0.422 |
| Platelet, cells/ul | 244.0(199.3,310.5) | 217.5(193.2,304.0) | 248.0(192.5,326.2) | 0.435 |
| AST, U/L | 93.25(33.00,208.30) | 171.10(64.55,283.83) | 59.50(25.55,152.65) | 0.015 |
| ALT, U/L | 82.25(33.77,133.12) | 122.15(79.87,231.80) | 45.75(25.50,113.97) | 0.007 |
| CK, U/L | 2967.5(649.5,6297.0) | 5475.00(3210.25,10410.75) | 1346.00(68.75,4860.75) | 0.001 |
| Variables | Total  (N=98) | Strong signal intensity  (n=34) | Weaker signal intensity  (n=64) | *P*-value |
| LDH, U/L | 538.5(199.0,892.0) | 408.00(209.75,782.00) | 530.50(118.75,1034.00) | <0.001 |
| LDL, mmol/L | 2.97(2.40,3.50) | 3.01(2.24,3.73) | 2.84(2.21,3.47) | 0.215 |
| HDL, mmol/L | 1.03(0.86,1.32) | 1.02(0.84,1.33) | 1.00(0.82,1.26) | 0.478 |
| elevated CRP, n/N (%) | 91/98(92.9%) | 29/34(85.3%) | 62/64(96.9%) | 0.034 |
| CRP, ng/ml | 7.05(3.02,24.29) | 2.95(2.14,20.62) | 11.02(5.37,19.15) | 0.001 |
| ESR, mm/h | 37.5(20.0,59.3) | 37.5(17.0,57.0) | 40.0(22.5,64.0) | 0.982 |
| IL6^b^, ng/ml | 6.0(3.0,10.0) | 7.0(4.0,14.5) | 5.5(3.0,8.7) | 0.065 |
| Ferritin^c^, ng/ml | 250.0(201.0,396.0) | 275.0 (150.5,420.5) | 215.0(130.5,351.5) | 0.187 |
| **Imaging finding** |  |  |  |  |
| NSIP, n/N (%) | 26/98(26.5%) | 10/34(29.4%) | 16/64(25.0%) | 0.748 |
| OP, n/N (%) | 6/98(6.1%) | 2/34(5.9%) | 4/64(6.3%) | 1.000 |
| NSIP+OP, n/N (%) | 38/98(38.8%) | 12/34(35.3%) | 26/64(40.6%) | 0.950 |
| **Pulmonary function tests** |  |  |  |  |
| FVC ^d^, % | 87.0(77.0,100.9) | 88.0(78.0,101.8) | 87.5(74.5,101.7) | 0.669 |
| FEV1 ^e^, % | 86.9(70.5,96.3) | 89.0(70.7,96.5) | 86.8(68.9,99.7) | 0.644 |
| FEV1/FVC ^f^ | 82.0(76.2,86.8) | 83.3(77.3,86.7) | 81.5(76.9,85.3) | 0.410 |
| DLCO ^g^, % | 82.0(69.8,89.1) | 84.5(76.3,88.8) | 78.4(63.5,89.4) | 0.288 |
| Follow-up duration, months | 36.0(17.5,60.0) | 24.0(15.5,54.0) | 37.0(13.7,49.8) | 0.064 |
| Duration from onset of symptoms to diagnosis, months | 5.0(2.0,10.0) | 4.0(2.0,11.5) | 5.5(2.3,12.0) | 0.077 |

Abbreviation: mRS, modified Rankin Scale; scores > 2 indicate moderate to severe disability; ILD, interstitial lung disease; RPILD, rapidly progressive interstitial lung disease; CTDs, connective tissue diseases; ANA, antinuclear antibody; RF, rheumatoid factor; AST, aspartate aminotransferase; ALT, alanine aminotransferase; CK, creatine kinase; LDH, lactate dehydrogenase; LDL, low-density lipoprotein; HDL, high-density lipoprotein; CRP, C reactive protein; ESR, erythrocyte sedimentation rate; IL6, interleukin-6; NSIP, non-specific interstitial pneumonia; OP, organising pneumonia; DLCO, percent predicted diffusing lung capacity for carbon monoxide; FEV1/FVC, forced breathing volume in the 1 s/forced vital capacity.

^a^ Other CTDs include rheumatic arthritis, systemic lupus erythematous, systemic sclerosis and Sjögren's syndrome.

^b^ IL6 data available for 82 patients.

^c^ Ferritin data available for 83 patients.

^d^ FVC data available for 65patients.

^e^ FEV1 data available for 66 patients.

^f^ FEV1/FVC data available for 66 patients.

^g^ DLCO data available for 66 patients.

**Table S2. Distribution of causes of death across the three clusters.**

| Cause of death | Cluster 1 (n=5) | Cluster 2 (n=2) | Cluster 3 (n=15) | Total  (n=22) |
| --- | --- | --- | --- | --- |
| Respiratory failure – RPILD | 0 | 1 | 2 | 3 |
| Respiratory failure – infection superimposed on ILD | 0 | 1 | 9 | 10 |
| Respiratory failure – COVID-19 (infection-related) | 1 | 0 | 2 | 3 |
| Cardio-cerebrovascular diseases | 2 | 0 | 1 | 3 |
| Lung cancer | 1 | 0 | 0 | 1 |
| Diabetic nephropathy | 0 | 0 | 1 | 1 |
| Hepatic failure | 1 | 0 | 0 | 1 |

Abbreviation: ILD, interstitial lung disease; RPILD, rapidly progressive interstitial lung disease.

**Table S3.** Clinical characteristics of 9 patients with IMNM whose muscle samples were subjected to proteomics analysis.

| **Patient** | **Group** | **Sex** | **Onset age(y)** | **Fever** | **Dyspnoea** | **ILD** | **RPILD** | **Respiratory failure** | **Muscle weakness** | **CK(U/L)** | **LDH(U/L)** | **Antibody** |
| --- | --- | --- | --- | --- | --- | --- | --- | --- | --- | --- | --- | --- |
| 1 | Cluster1 | Female | 56 | No | Yes | Yes | No | No | Yes | 4051 | 807 | SRP |
| 2 | Cluster3 | Female | 47 | Yes | Yes | Yes | No | Yes | Yes | 2142 | 560 | SRP |
| 3 | Cluster1 | Female | 56 | No | No | No | No | No | Yes | 6867 | 1401 | SRP |
| 4 | Cluster1 | Male | 52 | No | No | No | No | No | No | 3366 | 1451 | SRP |
| 5 | Cluster1 | Female | 56 | No | No | No | No | No | No | 375 | 85 | SRP |
| 6 | Cluster2 | Female | 25 | No | Yes | No | No | No | No | 14428 | 1966 | SRP |
| 7 | Cluster2 | Male | 68 | Yes | No | No | No | No | Yes | 2004 | 362 | HMGCR |
| 8 | Cluster3 | Female | 60 | Yes | No | No | No | No | Yes | 3714 | 533 | HMGCR |
| 9 | Cluster3 | Female | 66 | Yes | Yes | Yes | Yes | No | Yes | 3796 | 1380 | HMGCR |

ILD, interstitial lung disease; RPILD, rapidly progressive interstitial lung disease; CK, creatine kinase; LDH, lactate dehydrogenase;

**Reference**

1. Xiao Y, Xie S, Li HD, Liu Y, Zhang H, Zuo X, et al. Characterised intron retention profiles in muscle tissue of idiopathic inflammatory myopathy subtypes. *Ann Rheum Dis* (2024) 83(7):901-14. Epub 20240612. doi: 10.1136/ard-2023-225035. PubMed PMID: 38302260.

2. Zhang X, Smits AH, van Tilburg GB, Ovaa H, Huber W, Vermeulen M. Proteome-wide identification of ubiquitin interactions using UbIA-MS. *Nat Protoc* (2018) 13(3):530-50. Epub 20180215. doi: 10.1038/nprot.2017.147. PubMed PMID: 29446774.

3. Langfelder P, Horvath S. WGCNA: an R package for weighted correlation network analysis. *BMC bioinformatics* (2008) 9:559. Epub 20081229. doi: 10.1186/1471-2105-9-559. PubMed PMID: 19114008; PubMed Central PMCID: PMCPMC2631488.

4. Yu G, Wang LG, Han Y, He QY. clusterProfiler: an R package for comparing biological themes among gene clusters. *Omics* (2012) 16(5):284-7. Epub 20120328. doi: 10.1089/omi.2011.0118. PubMed PMID: 22455463; PubMed Central PMCID: PMCPMC3339379.
